# Exploring the Effects of *bolA* in Biofilm Formation and Current Generation by *Shewanella oneidensis* MR-1

**DOI:** 10.3389/fmicb.2020.00815

**Published:** 2020-05-08

**Authors:** Ana V. Silva, Miriam Edel, Johannes Gescher, Catarina M. Paquete

**Affiliations:** ^1^Instituto de Tecnologia Química e Biológica António Xavier, Universidade Nova de Lisboa, Oeiras, Portugal; ^2^Department of Applied Biology, Institute for Applied Biology, Karlsruhe Institute of Technology, Karlsruhe, Germany; ^3^Institute for Biological Interfaces, Karlsruhe Institute of Technology, Karlsruhe, Germany

**Keywords:** *bolA*, biofilms, microbial electrochemical technologies, bioelectrochemical systems, transcription factor, electroactive organisms

## Abstract

Microbial electrochemical technologies (METs) have emerged in recent years as a promising alternative green source of energy, with microbes consuming organic matter to produce energy or valuable byproducts. It is the ability of performing extracellular electron transfer that allows these microbes to exchange electrons with an electrode in these systems. The low levels of current achieved have been the limiting factor for the large-scale application of METs. *Shewanella oneidensis* MR-1 is one of the most studied electroactive organisms regarding extracellular electron transfer, and it has been shown that biofilm formation is a key factor for current generation. The transcription factor *bolA* has been identified as a central player in biofilm formation in other organisms, with its overexpression leading to increased biofilm. In this work we explore the effect of this gene in biofilm formation and current production by *S. oneidensis* MR-1. Our results demonstrate that an increased biofilm formation and consequent current generation was achieved by the overexpression of this gene. This information is crucial to optimize electroactive organisms toward their practical application in METs.

## Introduction

Microorganisms have developed strategies to live in virtually every environment on Earth, being able to scavenge energy from a wide range of organic and inorganic compounds. Among these, electroactive organisms harvest energy by reducing insoluble terminal electron acceptors, such as metal oxides in their natural environment or electrodes in microbial electrochemical technologies (METs) (Richardson, 2000; Kracke et al., 2015). In the last decades, these organisms have received considerable attention due to their promising role as self-regenerating catalysts in bioelectrochemical systems (BES) to convert chemical energy stored in biodegradable substances into electrical current (Logan et al., 2006) and added-value compounds (Rabaey and Rozendal, 2010). Although BES arose as a sustainable platform for electricity production (Logan et al., 2006), nowadays they can also be used for wastewater treatment, environmental bioremediation, biofuel production, bioelectrosynthesis and biosensing (Logan and Regan, 2006; Arends and Verstraete, 2012; Ucar et al., 2017; Logan et al., 2019; Mohan et al., 2019; Simonte et al., 2019). However, their large-scale application has been set back by the low power densities obtained so far, mainly limited by the slow electron transfer rates between electroactive organisms and electrodes (Logan and Rabaey, 2012; Logan et al., 2019). Toward this, numerous efforts have been made in recent years to optimize power generation in BES, including the characterization and optimization of electroactive organisms, in particular of the model organisms *Shewanella oneidensis* MR-1 and *Geobacter sulfurreducens* (Teravest et al., 2015; Yang et al., 2015; Bursa et al., 2017; Min et al., 2017; Ueki et al., 2018; Delgado et al., 2019; Fonseca et al., 2019; Reguera and Kashefi, 2019).

In BES, electroactive organisms interact directly with the electrode, via outer membrane cytochromes, nanowires or pili, or indirectly, using redox mediator molecules (e.g., flavins) (Costa et al., 2018; Edel et al., 2019). At the electrode, they form electroactive biofilms (EABs) (Kalathil et al., 2013), that can be diverse in terms of thickness, conductivity and shape. Indeed, the thickness and the composition of the biofilm can significantly increase current generation in BES. The increase in biofilm formation by methods such as protein overexpression (Liu et al., 2015), artificial production of biofilms by exoelectrogen immobilization (Yu et al., 2011), self-assembled graphene oxide – *S. oneidensis* MR-1 biofilm (Yong et al., 2014) and 3D printing (Zajdel et al., 2018; Freyman et al., 2019) lead to significant increases in current densities, highlighting the potential of EAB manipulation to optimize BES.

The *bolA* gene, initially identified as a transcriptional regulator for cell shape in *Escherichia coli* (Aldea et al., 1988), was recently shown to be responsible for promoting biofilm development by targeting several genes encoding proteins related to this process (Dressaire et al., 2015). This gene is present in numerous electroactive organisms (Supplementary Table S1), including in *S. oneidensis* MR-1. Herein, we report the effect of the *bolA* gene in the development of *S. oneidensis* MR-1 biofilm and the consequences for current density production in BES. We demonstrate that the *bolA* gene increases both current generation in BES and biofilm formation, opening the way for future regulation studies focusing on this gene. This work is a step forward towards a clear understanding of the genes that regulate biofilm formation in *S. oneidensis* MR-1. This knowledge is of great importance towards the optimization of current production in BES, paving the way for their commercial application.

## Materials and Methods

### Construction of an *S. oneidensis* MR-1 *bolA* Knock-Out Strain

The *bolA* knock-out strain (Table 1) was constructed according to what is described in Saltikov and Newman (2003). Briefly, regions of 500 bp up- and downstream of the *bolA* gene (SO_1099) (primers 1–4 Supplementary Table S2) were amplified via polymerase chain reaction (PCR). The resulting up- and downstream regions were inserted into the *Bam*HI and *Sal*I cleaved suicide vector pMQ150 and transformed into *E. coli* WM3064. This was later transferred to *S. oneidensis* MR-1 by conjugation. *S. oneidensis* MR-1 transconjugants containing the integrated mutagenesis vector were selected on Luria Bertani broth (LB) agar plates supplemented with kanamycin (50 μg/mL). The colonies were grown in the absence of antibiotic and subcultured twice into LB. After overnight growth at 30°C, dilutions were plated on LB agar plates supplemented with 10% sucrose and incubated overnight at 30°C. Plates were replica printed onto LB plates and LB plates supplemented with kanamycin (50 μg/mL), and kanamycin-sensitive colonies were screened by PCR for the selection of the strains with the deletion of *bolA* gene (primers 5, 6 Supplementary Table S2).

**TABLE 1:** Bacterial strains used in this study.

**Name**	**Strain**
WT	*S. oneidensis* MR-1 with empty pBBR1MCS-2
WT+	*S. oneidensis* MR-1 harboring pBBR1MCS-2_*bolA*
Δ*bolA*	*S. oneidensis* MR-1 Δ*bolA* with empty pBBR1MCS-2
Δ*bolA*+	*S. oneidensis* MR-1 Δ*bolA* harboring pBBR1MCS-2_*bolA*

### Cloning the *bolA* Gene

DNA fragments containing the *bolA* gene from *S. oneidensis* MR-1 were amplified via PCR from genomic DNA using the primers 9 and 10 listed in Supplementary Table S2. In order to include a ribosome binding site (RBS), the gene was first amplified with the primers containing the RBS sequence (7, 8 Supplementary Table S2). The PCR product was then inserted into the pBBR1MCS-2 vector, previously digested with the restriction enzymes *Eco*RV and *Bam*HI (NZYTech, Portugal), using NEBuilder HiFi DNA Assembly Cloning kit (New England Biolabs). The vector containing the *bolA* gene was then transformed into the strains *S. oneidensis* MR-1 and *S. oneidensis* MR-1 Δ*bolA*, creating the strains WT+ and Δ*bolA*+, respectively (Table 1). The vector pBBR1MCS-2 was a gift from Kenneth Peterson (Addgene plasmid # 85168) (Kovach et al., 1995).

### Bacterial Growth Conditions

Bacterial strains were cultivated overnight in LB supplemented with kanamycin (50 μg/mL) at 30°C with 150 rpm agitation. For the growth curves, cells were diluted in M4 medium (Delgado et al., 2019) supplemented with kanamycin (50 μg/mL) to a starting OD_600_ of 0.07. 200 μL were then transferred to individual wells of a polystyrene flat bottom 96-well plate (Sarstedt) and allowed to grow at 30°C without stirring. The cell density was measured at 600 nm every 30 min for 46 h using a Multiskan Sky Microplate Spectrophotometer (Thermo Scientific^TM^). The experiment was conducted using twelve replicates and the mean and standard deviation were calculated using MS Excel.

### Microscopic Imaging

Planktonic cells were harvested from the cultures after the growth curves (46 h of growth). Samples were observed in slides coated with 1.5% (wt/vol) agarose film and enclosed with a cover glass. Images were acquired on a Leica DM 6000B upright microscope equipped with an Andor iXon 885 EMCCD camera and controlled with the MetaMorph V5.8 software, using the 100 × 1.4 NA oil immersion objective plus a 1.6× optvar. Images were processed using ImageJ software.

### Biofilm Characterization

Growth and analysis of static biofilms under microoxic conditions were measured using crystal violet staining on the 96-well plate resulting from the growth curve experiment according to (Dressaire et al., 2015). Each well was cleaned three times with 200 μL water and then treated with 200 μL 0.1% crystal violet for 15 min. The plate was then washed three times with water to remove the excess of crystal violet and dried at 65°C for 15 min. To solubilize the crystal violet, 200 μL of 96% ethanol were added to each well and incubated for 15 min at room temperature and the optical density of each well was measured at 570 nm using a Multiskan Sky Microplate Spectrophotometer (Thermo Scientific^TM^). The ratio of biofilm development to planktonic growth was calculated using the cell density (OD_570_/OD_600_). An unpaired *t*-test was used to determine significance of the data. The level of significance was set to 5%.

### Bioelectrochemical Systems

The bioelectrochemical experiments were conducted in triplicates using a single chamber BES with a working volume of 270 mL (Bursa et al., 2017). Graphite felt (projected area of 36 cm^2^, SGL Group, Germany) and platinum mesh (projected area of 1.25 cm^2^, chemPUR, Germany) were used as working and counter electrode material, respectively, and an Ag/AgCl electrode [sat. KCl, 0.199 V vs standard hydrogen electrode (SHE)] (Sensortechnik Meinsberg, Germany) was used as reference electrode. Before use, the working electrode was rinsed with isopropanol, followed by deionized water. The complete bioelectrochemical setup was sterilized by autoclaving.

Before inoculation, cells were harvested from the preculture by centrifugation (7 min, 6000 × *g*) and washed 3 times with M4 medium containing neither electron donor nor electron acceptor. Cells were then resuspended to a starting OD_600_ of 0.07 in M4 medium containing 70 mM lactate and kanamycin (50 μg/mL).

During chronoamperometric experiments, the working electrode was poised to 0 mV vs SHE and current was monitored for 46 h. BES were incubated at 30°C and constantly flushed with N_2_ gas in order to ensure anoxic conditions. For constant mixing of the liquid phase, the medium was continuously agitated using a magnetic stirrer. The OD_600_ was measured in the beginning and after the 46 h of the BES experiments and showed that the planktonic growth was negligible (ΔOD_600_ < 0.02).

An unpaired *t*-test was used to determine significance of the data. The level of significance was set to 5%.

### DNA Isolation and qPCR

For DNA Isolation, the innuPREP Stool DNA Kit (Analytic Jena, Germany) was used according to the manufacturers’ suggestions with minor modifications. Relative cell quantifications of anode sample are based on three individual BES. Anodes from the BES were sliced into small pieces and 5 mL of SLS buffer were added. The samples were vortexed vigorously for 1 min. Thereafter, the samples were incubated at 95°C for 15 min, and vortexed every 5 min. DNA isolation proceeded according to the manufacturer’s protocol. The quantitative PCR was performed in duplicate for each sample. Considering the triplicates of the BES experiments, the PCR result for each of the strains is based on the analysis of six samples. Quantitative PCR was conducted using Primers 11 and 12 (Supplementary Table S2) according to (Dolch et al., 2014).

For quantitative analysis, a standard curve using biological triplicates of the bacterial strains in six different dilutions was established. Before isolation of the DNA, the cells were counted in two different dilutions (Neubauer chamber improved, Friedrichsdorf, Germany). Based on the standard curves, cell counts of isolated DNA sample were determined. An unpaired *t*-test was used to determine significance of the data. The level of significance was set to 5%.

## Results

### Bacterial Growth and Cell Shape

**FIGURE 1:**
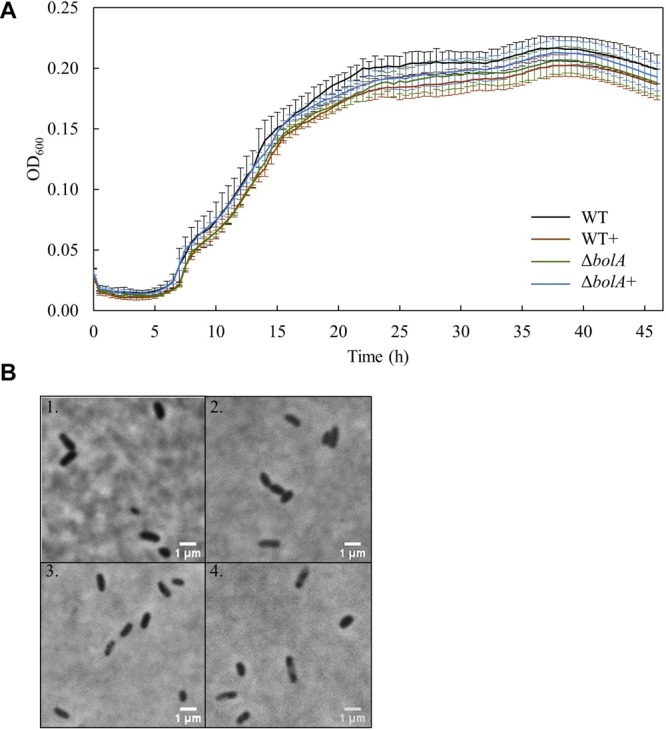
**(A)** Growth curves of the different *S. oneidensis* MR-1 strains obtained in the 96 well-plate: Black - WT; Brown – WT+; Green – Δ*bolA*; Blue – Δ*bolA*+. Cells were cultured for 46 h under microaerobic conditions in M4 medium and OD_600_ was measured every 30 min. **(B)** Phase contrast microscope imaging of the different strains: 1 – WT; 2 – WT+; 3 – Δ*bolA*; 4 – Δ*bolA*+. Pictures were taken using agarose-coated slides and cultures resulting from the growth curves.

Bacterial growth profile of the different strains was monitored for 46 h under microoxic conditions to evaluate the effect of *bolA* (Figure 1A). There were no significant differences detected in the growth of the strains. Furthermore, the microscopic images taken to explore differences in cell shape show that no morphological differences exist between the strains at this scale (Figure 1B).

### Biofilm Formation Under Static Conditions

**FIGURE 2:**
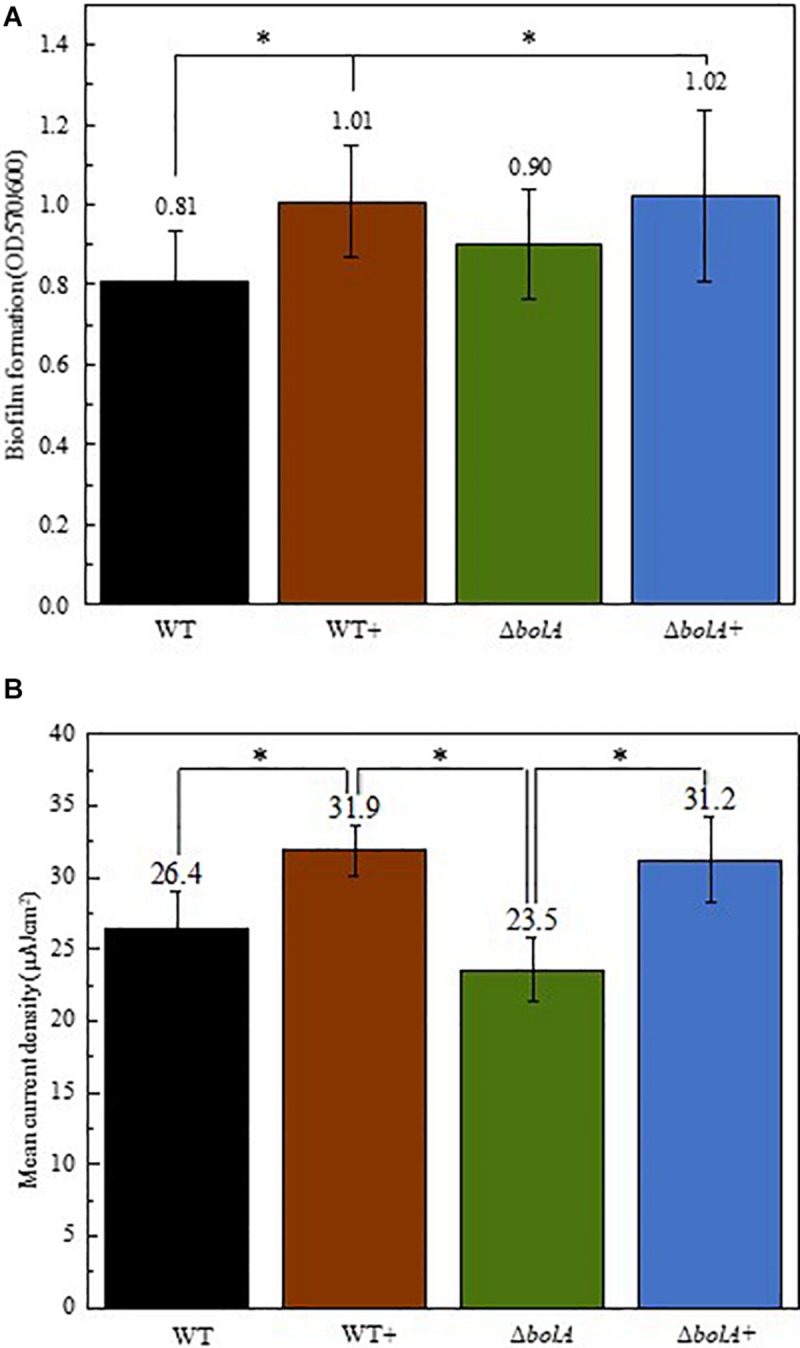
Representation of the biofilm production by the different strains normalized by OD_600_ of the planktonic cultures **(A)** and mean current density produced by the different strains in BES **(B)**. Black – WT; Brown – WT+; Green – Δ*bolA*; Blue – Δ*bolA*+. Biofilm formation was measured by crystal violet stain with cell cultures resulting from the growth under microaerobic conditions after 46 h incubation. Error bars represent standard deviations. The potential of the anode in BES was poised to 0 vs SHE using an Ag/AgCl reference electrode. Stars represent significant differences (unpaired *t*-test *p* < 0.05).

Cultures resulting from the growth curve were used to evaluate biofilm production by crystal violet staining (Figure 2A). The results show that the overexpression of *bolA* leads to an increase in biofilm production, with significant differences (unpaired *t*-test *p* < 0.05) found between the WT and both the WT+ and Δ*bolA*+. The strain WT+ produces a 1.24-fold increase in the ratio of planktonic cells to biofilm relative to the WT while the strain Δ*bolA*+ produces a 1.26-fold increase. Although the Δ*bolA* strain presents a slight increase in biofilm formation relative to the WT strain this difference is not significant.

### Bioelectrochemical Systems

The effect of the deletion and overexpression of *bolA* in current density was explored in BES. The curves of the current density of the WT and the Δ*bolA*, as well as of the WT+ and the Δ*bolA*+ present very similar profiles (Supplementary Figure S1). When comparing the WT with the WT+ and the Δ*bolA* with the Δ*bolA*+, an increase in the produced current density can be observed. Indeed, the mean current density produced for the WT strain upon *bolA* expression (Figure 2B) shows a significantly higher current generation (unpaired *t*-test *p* < 0.05) with a 1.2-fold increase. A very similar effect can be observed in the Δ*bolA*+ strain.

### Quantitative PCR (qPCR)

**FIGURE 3:**
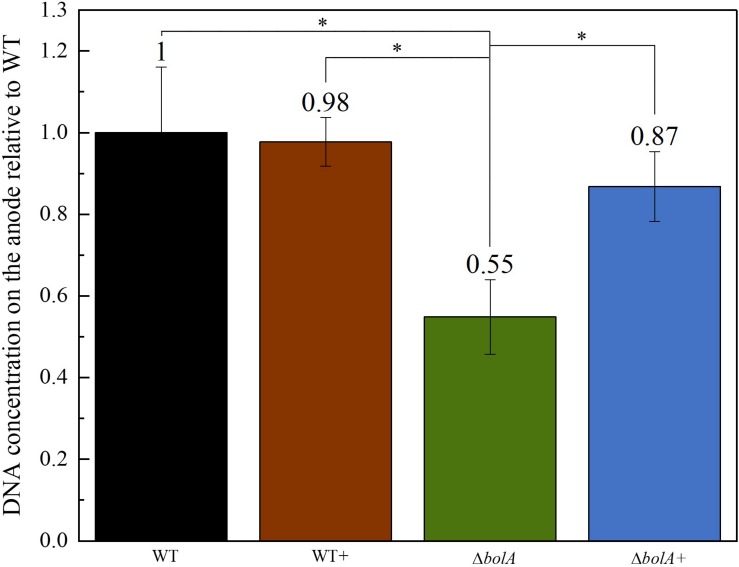
Amount of DNA on the anode relative to the wildtype determined by qPCR. DNA was isolated from the anodes after BES experiments. Stars represent significant differences (unpaired *t*-test *p* < 0.05).

The DNA deposited on the anodes of BES was isolated using qPCR and the DNA content was examined to evaluate if the increased current density of the *bolA* overexpressing strains correlates with cell attachment on the anode. Interestingly, the deletion of *bolA* leads to a 1.8-fold decreased amount of DNA on the anode (Figure 3), while the complementation of the *bolA* deletion shows almost the same amount of DNA on the anode as the WT. No significant difference was observed between the WT and the *bolA* overexpressing strain (WT +).

## Discussion

The *bolA* gene was originally identified as a transcriptional regulator for cell shape in *E. coli*, as its overexpression leads to round cell morphology (Aldea et al., 1988). BolA was demonstrated to promote biofilm development by targeting several genes encoding proteins related to this process, being described as a modulator for the switch between planktonic to sessile lifestyles (Vieira et al., 2004; Dressaire et al., 2015).

In this work we addressed the effect of this gene in *S. oneidensis* MR-1, one of the most widely studied microorganism regarding extracellular electron transfer in BES. It has been demonstrated that, in these systems, an increase in biofilm thickness leads to an improvement in current generation (Yu et al., 2011; Yong et al., 2014; Liu et al., 2015; Zajdel et al., 2018; Freyman et al., 2019).

**FIGURE 4:**
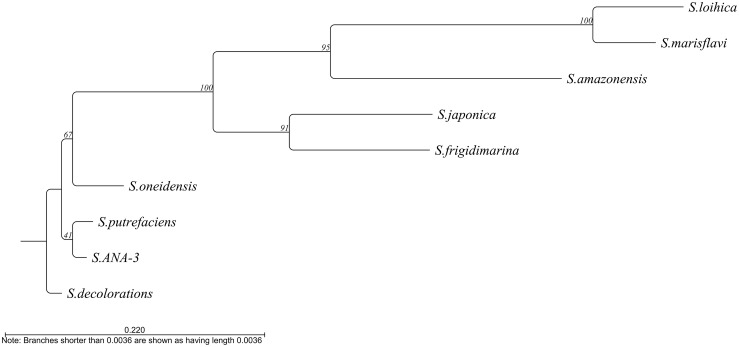
Phylogenetic tree based on the alignment of the *bolA* sequence from the different electroactive *Shewanella* species. Tree constructed by neighbor joining using CLC main workbench 8.1 software (Qiagen).

At least 10 of the 11 *Shewanella* species described as electroactive (Koch and Harnisch, 2016) contain this gene. The only exception is *S. electrodiphila* for which the genome is not available and, therefore, the presence of the gene could not be confirmed. Among the different electroactive *Shewanella* species, the gene maintains 39.62% identity, with *S. lohica* being the least similar to *S. oneidensis* MR-1 (56.12% identity) and *S.* sp. ANA-3 the most similar (93.4% identity) (Figure 4).

In accordance to what is described for *E. coli* (Dressaire et al., 2015) and *Salmonella enterica* (Mil-homens et al., 2018), the overexpression or deletion of *bolA* does not affect the growth profiles of the different strains. Moreover, contrary to what is described for *E. coli*, we could not detect any cell shape alteration in *S. oneidensis* MR-1 upon *bolA* overexpression (Figure 1B). Although the stress-inducible morphogene *bolA* is widely conserved in all kingdoms of life, leading to a decrease of surface to volume ratio as an adaptation to harsh conditions (Guinote et al., 2014), it does not affect cell shape in carbon starvation or late stages of growth in all organisms (Koch and Nybroe, 2006). Indeed, no changes in cell morphology could be detected upon *bolA* overexpression in *Pseudomonas fluorescens* (Koch and Nybroe, 2006), a bacterium that is highly homologous to *S. oneidensis* MR-1 (Hau and Gralnick, 2007). Furthermore, it has been shown that only some homologs of the *bolA* gene have an effect on cell morphology in *E. coli* (Khona et al., 2013).

Differences could also be observed in the biofilm formation, with the overexpression and complemented strains producing an approximate 1.25-fold increase in biofilm production. This result relates very well with the 1.2-fold increase in the mean current density observed upon expression of the *bolA* gene. Although, the amount of DNA deposited on the anode in the Δ*bolA*+ strain was higher than that of the Δ*bolA*, this was not observed for the WT strain. As the qPCR detects both intracellular DNA and extracellular DNA, that can be embedded in the biofilm matrix and help in extracellular electron transfer processes, it is difficult to determine if the *bolA* gene promotes biofilm formation by increasing extracellular DNA.

Our data corroborates the hypothesis that an increase in biofilm thickness can lead to higher current generation by electroactive organisms (Yu et al., 2011; Yong et al., 2014; Liu et al., 2015; Zajdel et al., 2018; Edel et al., 2019; Freyman et al., 2019). The deletion of *bolA*, however, does not interfere with biofilm formation, with the strain producing comparable amounts of biofilm to the WT. This may be a consequence of the fact that the harsh conditions necessary for *S. oneidensis* MR-1 to activate BolA have not been achieved in the WT strain. The same results were reported for *E. coli* but have not been explored further (Dressaire et al., 2015).

The biofilm assay was conducted under microoxic conditions given the inability to detect biofilm formation using this protocol under anoxic conditions. This could be attributed to the low optical densities obtained when growing *S. oneidensis* MR-1 in minimal medium under anoxic condition in the 96-well plates. Moreover, in the case of oxic or microoxic conditions, the biofilm is formed on the top of the well, at the interface between the culture medium and oxygen, which allows the cells to get both nutrients and the terminal electron acceptor (i.e., oxygen). In contrast, under anoxic conditions, this interface is not present, as the terminal electron acceptor (i.e., fumarate) is soluble in the medium. In BES, the oxygen is absent and the electrode acts as the terminal electron acceptor, promoting biofilm development on its surface.

This work demonstrates that *bolA* is involved in the biofilm formation in *S. oneidensis* MR-1, with its overexpression contributing to an increase in biofilm formation. We could also demonstrate that this increase enhances current production in BES. Further studies are, however, necessary to characterize the regulatory network of this gene and its protein product. Indeed, it has been demonstrated that *bolA* interfere with the transition from planktonic lifestyle to biofilm by balancing the intracellular concentration of cyclic-di-GMP (Moreira et al., 2017). Interestingly, this bacterial second messenger was also shown to be an important intracellular regulator for controlling biofilm stability in *S. oneidensis* MR-1 (Thormann et al., 2006). The quantification of cyclic-di-GMP, as well as the understanding of what processes *bolA* regulates would increase our knowledge in enhancing biofilm formation and consequently increase current production in BES.

## Data Availability Statement

The datasets generated for this study are available on request to the corresponding author.

## Author Contributions

AS and ME performed the experiments and carried out the data acquisition and analysis. AS and CP drafted the manuscript. All authors were involved in the discussion of the results and in writing the manuscript. CP and JG revised the manuscript. All authors approved the final manuscript.

## Conflict of Interest

The authors declare that the research was conducted in the absence of any commercial or financial relationships that could be construed as a potential conflict of interest.
