# Supplementary material for: Exploring the Effects of bolA in Biofilm Formation and Current Generation by Shewanella oneidensis MR-1
Source: Front Microbiol. 2020 May 8;11:815. doi: 10.3389/fmicb.2020.00815 (PMC7225295; doi:10.3389/fmicb.2020.00815)
Supplement: Supplementary file 1 [file Data_Sheet_1.docx]

Supplementary Material

# Primers used

**Table S1:** List of electroactive organisms (according to Koch and Harnisch 2016) with indication of the presence or absence of the BolA protein. The presence of BolA was determined by searching each organisms’ genome in NCBI with the term “BolA” in the corresponding protein list.

| Organism | BolA presence | BolA code |
| --- | --- | --- |
| *Acidiphilium cryptum* | Yes | WP_007423540.1 |
| *Acidithiobacillus ferrooxidans* | Yes | WP_009560822.1 |
| *Acinetobacter calcoaceticus* | Yes | WP_133975907.1 |
| *Acinetobacter johnsonii* | Yes | WP_005401913.1 |
| *Actinobacillus succinogenes* | Yes | WP_012072352.1 |
| *Aeromonas hydrophila* | Yes | WP_010673364.1 |
| *Anabaena variabilis* | Yes | WP_010994972.1 |
| *Azospira suillum* | Yes* | [WP_014236502.1/WP_043799444.1](https://www.ncbi.nlm.nih.gov/protein/WP_109039550.1?report=genbank&log$=prottop&blast_rank=4&RID=GEY17KK8014) |
| *Bacillus subtilis* | Yes | WP_100506968 |
| *Branhamella catarrhalis* | Yes | WP_120672796.1 |
| *Brevundimonas diminuta* | Yes | WP_003166945.1 |
| *Burkholderia cepacia* | Yes | WP_059233936.1 |
| *Calditerrivibrio nitroreducens* | No | - |
| *Citrobacter freundii* | Yes | WP_038638264.1 |
| *Citrobacter sp.* SX-1 | Yes* | WP_042324511.1 |
| *Clostridium aceticum* | No | - |
| *Clostridium acetobutylicum* | No | - |
| *Clostridium butyricum* | No | - |
| *Clostridium ljungdahlii* | No | - |
| *Clostridium pasteurianum* | No | - |
| *Clostridium propionicum* | No | - |
| *Clostridium tyrobutyricum* | No | - |
| *Comamonas denitrificans* | Yes* | WP_043373767.1/WP_003058653.1 |
| *Comamonas testosteroni* | Yes | WP_034365915.1 |
| *Corynebacterium glutamicum (Brevibacterium flavum)* | No | - |
| *Corynebacterium sp.* MFC03 | No* | - |
| *Dechloromonas agitata* | Yes | WP_027458058.1 |
| *Desulfitobacterium hafniense* | No | - |
| *Desulfobulbus propionicus* | No | - |
| *Desulfovibrio desulfuricans* | No | - |
| *Desulfuromonas acetoxidans* | No | - |
| *Enterobacter cloacae (Erwinia dissolvens)* | Yes | WP_129362423.1 |
| *Escherichia coli* | Yes | NP_414969.4 |
| *Faecalibacterium prausnitzii* | No | - |
| *Geoalkalibacter ferrihydriticus* | No | - |
| *Geoalkalibacter subterraneus* | No* | - |
| *Geobacillus sp.* S2E | No* | - |
| *Geobacter anodireducens* | No* | - |
| *Geobacter bemidjiensis* | No | - |
| *Geobacter bremensis* | No | - |
| *Geobacter chapellei* | No* | - |
| *Geobacter humireducens* | No* | - |
| *Geobacter hydrogenophilus* | No* | - |
| *Geobacter lovleyi* | No | - |
| *Geobacter metallireducens* | No | - |
| *Geobacter sulfurreducens* | No | - |
| *Geobacter uraniireducens* | No | - |
| *Geopsychrobacter electrodiphilus* | No | - |
| *Geothrix fermentans* | No | - |
| *Gluconobacter oxydans* | Yes | WP_062449716.1 |
| *Kingella denitrificans* | Yes | WP_003781876.1 |
| *Kingella kingae* | Yes | WP_003785498.1 |
| *Klebsiella pneumoniae* | Yes | WP_048270466.1 |
| *Lactobacillus plantarum* | No | - |
| *Mariprofundus ferrooxydans* | Yes | WP_009849673.1 |
| *Methanobacterium palustre* | No* | - |
| *Methanococcus maripaludis* | No | - |
| *Micrococcus luteus* | No | - |
| *Moorella thermoacetica* | No | - |
| *Ochrobactrum anthropi* | Yes | WP_010657613.1/WP_006466620.1 |
| *Propionibacterium freudenreichii* | No | - |
| *Proteus mirabilis* | Yes | WP_088493680.1 |
| *Proteus vulgaris* | Yes | WP_072069384.1 |
| *Pseudomonas aeruginosa* | Yes | WP_009686014.1 |
| *Pseudomonas alcaliphila* | Yes | WP_074675136.1 |
| *Pseudomonas fluorescens* | Yes | WP_126365315.1 |
| *Raoultella electrica* | Yes | WP_041144317.1 |
| *Rhodobacter capsulatus* | Yes | WP_013066292.1 |
| *Rhodobacter sphaeroides* | Yes | YP_352030.1 |
| *Rhodoferax ferrireducens* | Yes | WP_011464683.1 |
| *Rhodopseudomonas palustris* | Yes | WP_110787197.1 |
| *Shewanella amazonensis* | Yes | WP_011760654.1 |
| *Shewanella decolorationis* | Yes | WP_023268362.1 |
| *Shewanella electrodiphila* | Yes* | WP_144044753.1/WP_144045332.1 |
| *Shewanella frigidimarina* | Yes | WP_011636419.1 |
| *Shewanella japonica* | Yes | WP_055023677.1 |
| *Shewanella loihica* | Yes | WP_011866680.1 |
| *Shewanella marisflavi* | Yes | WP_088904080.1 |
| *Shewanella oneidensis* | Yes | NP_716725.2 |
| *Shewanella putrefaciens* | Yes | WP_011621726.1 |
| *Shewanella sp.* ANA-3 | Yes | WP_011716063.1 |
| *Shewanella sp.* HN-41 | Yes | WP_037458006.1 |
| *Shigella flexneri* | Yes | NP_706328.3 |
| *Spirulina platensis* | Yes | WP_006622964.1 |
| *Sporomusa ovata* | No | - |
| *Sporomusa silvacetica* | No | - |
| *Sporomusa sphaeroides* | No | - |
| *Staphylococcus carnosus* | No | - |
| *Streptococcus lactis* | No | - |
| *Synechococcus* PCC 6301 | Yes | WP_011242716.1 |
| *Thermincola ferriacetica* | No | - |
| *Thermincola potens* | No | - |
| *Tolumonas osonensis* | Yes* | TXH65455.1/TXH61899.1 |
| *Winogradskyella poriferorum* | No* | - |
| * Genome of the organism could not be found. The indication of the presence of BolA was based on the genus information. | | |

**Table S2:** List of primers used in the study and their purpose.

| **Number** | **Name** | **Sequence** | **Purpose** |
| --- | --- | --- | --- |
| 1 | pMQSalI_500up | CAGTGCCAAGCTTGCATGCCTGCAGGTTGCAAAAATTACGCGATAAATTAC | Deletion of *bolA* |
| 2 | RevCom_500up | ATTCAAATCTCGACATGGGTAAATC |  |
| 3 | 500up_500dn | AGATTTGAAT TTAACGCGATTTAGGACGG |  |
| 4 | 500dn_pMQBamHI | CTGCCTTTGCGGTATTCG GATCCCCGGGTACCGAGCTCGAATTCG |  |
| 5 | Confirmation_ FW | GTATGGCGACCTAGGATGT | Test deletion of *bolA* |
| 6 | Confirmation_Rv | GAGTTTTGCCCTTCCAATAATAGG |  |
| 7 | RBS_bolA_Forw | TAAGAAGGAGATATACATCCCATGTCCAATACCCAAGAGCAGGGCACAGTG | Insert a ribosome binding site |
| 8 | RBS_bolA_Rev | ATGAATAATTTTATCAGATCCCATTTAACCGCGGCAATTAGGTGTTTTGGGC |  |
| 9 | bolA_pBBR_Forw | GTATCGATAAGCTTGATATCGAAGGAGATATACATACCC | Clone *bolA* in pBBR plasmid |
| 10 | bolA_pBBR_Rev | CTAGAACTAGTGGATCCTTAACCGCGGCAATTAG |  |
| 11 | *S. oneidensis* mioC_for_qPCR | TATTCAAGTGCTTCTATTAG | qPCR analysis |
| 12 | *S. oneidensis* mioC_rev_qPCR | AAGAACTTCTACTCAACA |  |


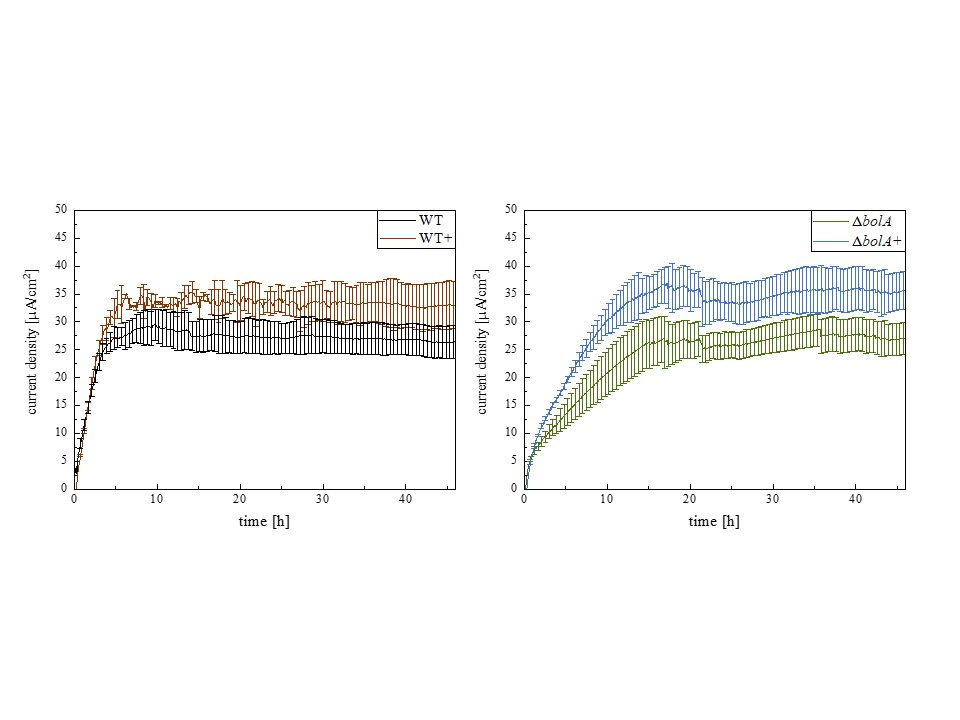


**Figure S1:** Current density produced by the different strains. Black- WT; Brown- WT+; Green- Δ*bolA*; Blue- Δ*bolA*+. The potential of the anode in BES was poised to 0 vs SHE using an Ag/AgCl reference electrode.
